# Phylogeny, Divergence Time Estimation and Biogeography of the Genus *Onnia* (Basidiomycota, Hymenochaetaceae)

**DOI:** 10.3389/fmicb.2022.907961

**Published:** 2022-07-07

**Authors:** Heng Zhao, Meng Zhou, Xiao-Yong Liu, Fang Wu, Yu-Cheng Dai

**Affiliations:** ^1^School of Ecology and Nature Conservation, Institute of Microbiology, Beijing Forestry University, Beijing, China; ^2^College of Life Sciences, Shandong Normal University, Jinan, China

**Keywords:** Hymenochaetaceae, molecular dating, reconstruct ancestral state, new taxa, biogeographic patterns

## Abstract

Species of *Onnia* are important tree pathogens and play a crucial role in forest ecosystems. The species diversity and distribution of *Onnia* have been studied, however, its evolutionary history is poorly understood. In this study, we reconstructed the phylogeny of *Onnia* using internal transcribed spacers (ITS) and large subunit (LSU) rDNA sequence data. Molecular clock analyses developed the divergence times of *Onnia* based on a dataset (ITS + LSU rDNA + *rpb1* + *rpb2* + *tef1*α). Reconstruct Ancestral State in Phylogenies (RASP) was used to reconstruct the historical biogeography for the genus *Onnia* with a Dispersal Extinction Cladogenesis (DEC) model. Here, we provide a robust phylogeny of *Onnia*, with a description of a new species, *Onnia himalayana* from Yunnan Province, China. Molecular clock analyses suggested that the common ancestor of *Onnia* and *Porodaedalea* emerged in the Paleogene period with full support and a mean stem age of 56.9 Mya (95% highest posterior density of 35.9–81.6 Mya), and most species occurred in the Neogene period. Biogeographic studies suggest that Asia, especially in the Hengduan-Himalayan region, is probably the ancestral area. Five dispersals and two vicariances indicate that species of *Onnia* were rapidly diversified. Speciation occurred in the Old World and New World due to geographic separation. This study is the first inference of the divergence times, biogeography, and speciation of the genus *Onnia*.

## Introduction

*Onnia* P. Karst. was proposed by Karsten and typified by *Onnia tomentosa* (Fr.) P. Karst. It is a homogeneous genus and forms a distinct clade in the Hymenochaetaceae based on phylogenetic analyses (Karsten, 1889; Wagner and Fischer, 2002; Larsson et al., 2006; Dai, 2010). Phylogenetically, *Onnia* is closely related to *Porodaedalea* Murrill, while morphologically *Porodaedalea* differs from *Onnia* by a perennial growth habit, pileate basidiocarps lacking a stipe, straight setae, and a dimitic hyphal system (Wagner and Fischer, 2002; Larsson et al., 2006; Dai, 2010; Ji et al., 2017). Almost all species of *Onnia* usually grow on gymnosperms, but one species, *Onnia vallata* (Berk.) Y.C. Dai and Niemelä, was recorded on angiosperms based on morphological features only and still without DNA data (Dai, 2010; Ryvarden and Melo, 2014; Ji et al., 2017). Some species of *Onnia* are well-known pathogens causing Tomentosus Root Rot on trees of Pinaceae, such as *Picea* and *Pinus* (Hunt and White, 1998; Germain et al., 2009; Ji et al., 2017).

Currently, eight species are accepted in *Onnia*, and their distribution is well defined. *Onnia tomentosa* is widespread in the Northern Hemisphere, including Canada, China, Czechia, Denmark, Finland, France, Germany, Italy, Norway, Poland, Russia, Spain, Sweden, the United Kingdom, Ukraine, and the United States (Table 1; Ryvarden and Gilbertson, 1993; Wagner and Fischer, 2001; Akata et al., 2009; Germain et al., 2009; Ji et al., 2017; Zhou and Wu, 2018; Wu et al., 2022). *Onnia leporina* (Fr.) H. Jahn is reported in Eurasia, such as China, Czechia, Finland, Italy, Norway, Sweden, and Ukraine (Ryvarden and Gilbertson, 1993; Wu et al., 2022). While *O. himalayana* Y.C. Dai, H. Zhao, and Meng Zhou, sp. nov., *O. microspora* Y.C. Dai and L.W. Zhou, and *O. tibetica* Y.C. Dai and S.H. He appear to be endemic to China, *O. kesiyae* M. Zhou and F. Wu, *O. subtriquetra* Vlasák and Y.C. Dai, and *O. triquetra* (Pers.) Imazeki occurred in Vietnam, the United States, and Europe (such as Czechia, Finland, France, Germany, Hungary, Poland, Russia, Spain, and Ukraine), respectively (Ryvarden and Gilbertson, 1993; Wu et al., 2022). Moreover, species of *Onnia* possessed host trees preferences, *O. tomentosa* and *O. leporina* mainly occurred on *Picea*, while other species commonly grow on *Pinus* (Ji et al., 2017; Wu et al., 2022). Indeed, species diversification and evolution of *Onnia* seem to inextricably interact with host trees and geographic separation, which provided niches for *Onnia* (Krah et al., 2018).

**TABLE 1:** Taxa information and GenBank accession numbers used in this study.

Species	Sample	GenBank accession nos.					Country
			
		ITS	LSU rDNA	*rpb1*	*rpb2*	*tef1*α	
*Amylocorticium cebennense*	HHB 2808	GU187505	GU187561	GU187439	GU187770	GU187675	United States
*Anomoloma myceliosum*	MJL 4413	GU187500	GU187559	GU187441	GU187766	GU187677	Canada
*Athelia arachnoidea*	CBS 418.72	GU187504	GU187557	GU187436	GU187769	GU187672	Netherlands
*Auricularia heimuer*	Xiaoheimao	LT716074	KY418890	KY418982	KY419035	KY419083	China
*Boletopsis leucomelaena*	AFTOL 1527	DQ484064	DQ154112	GU187494	GU187820	GU187763	United States
*Bondarzewia montana*	AFTOL 452	DQ200923	DQ234539	DQ256049	AY218474	DQ059044	Canada
*Coltricia perennis*	Cui 10318	KU360686	KJ000224	–	–	–	China
*Cryptococcus humicola*	AFTOL 1552	DQ645516	DQ645514	–	DQ645517	DQ645519	–
*Dacryopinax spathularia*	AFTOL 454	AY854070	AY701525	–	AY786054	AY881020	–
*Fomitiporia hartigii*	MUCL 53551	JX093789	JX093833	–	JX093877	JX093746	Estonia
*F*. *langloisii*	MUCL 46375	EF429242	EF429225	–	–	–	United States
*F*. *mediterranea*	AFTOL 688	AY854080	AY684157	–	AY803748	AY885149	
*Gloeophyllum sepiarium*	Wilcox-3BB	HM536091	HM536061	–	HM536109	HM536110	United States
*Gomphidius roseus*	MB 95-038	DQ534570	DQ534669	GU187459	GU187818	GU187702	Germany
*Grifola frondosa*	AFTOL 701	AY854084	AY629318	AY864876	AY786057	AY885153	
*Gymnopilus picreus*	ZRL2015011	LT716066	KY418882	KY418980	KY419027	KY419077	China
*Hydnoporia lamellata*	Cui 7629	JQ279603	JQ279617	–	–	–	China
*Inonotus griseus*	Dai 13436	KX364802	KX364823	KX364871	KX364919	MF977775	China
*Jaapia argillacea*	CBS 252.74	GU187524	GU187581	GU187463	GU187788	GU187711	Netherlands
*Lepiota cristata*	ZRL20151133	LT716026	KY418841	KY418963	KY418992	KY419048	China
*Leptosporomyces raunkiaeri*	HHB 7628	GU187528	GU187588	GU187471	GU187791	GU187719	United States
*Neurospora crassa*	OR74A	HQ271348	AF286411	–	AF107789	XM959775	–
*Onnia kesiyae*	Dai 18415	NR_160600	NG_068811	–	–	**OM800827**	Vietnam
*Onnia leporina*	Dai 13501	KT281958	–	–	–	–	China
	Dai 20866	**OM677245**	**OM677252**	–	–	**OM800829**	China
	JV0609/15	KT281959	–	–	–	–	Czechia
	JV1207/2	KT281960	KT281972	–	–	–	Czechia
	Phaeo1	KF996514	–	–	–	–	Italy
*Onnia microspora*	Dai 11886	KT281956	KT281970	–	–	–	China
	Dai 11897	KT281957	KT281971	–	–	–	China
*Onnia himalayana*	Dai 22620	**OM677247**	**OM677254**	–	–	–	China
*Onnia subtriquetra*	Dai 23686	**OM677244**	**OM677251**	**ON007276**	**OM937018**	**OM800828**	United States
	Dai 23687	OM967274	OM967335	–	–	–	United States
	MB2	KT281955	KT281969	–	–	–	United States
	JV0410/12J	KT281954	KT281968	–	–	–	United States
	JV0109/D6J	KT281953	KT281967	–	–	–	United States
*Onnia tibetica*	Cui 12254	KT281961	KT281973	–	–	–	China
	Dai 23621	OM967275	OM967336	–	–	–	China
	Dai 23622	–	OM967337	–	–	–	China
	Dai 23642	**OM677246**	**OM677253**	**ON007277**	**OM937019**	**OM800830**	China
	Dai 23643	OM967276	OM967338	–	–	–	China
	Yuan 1964	KT281962	KT281974	–	–	–	China
*Onnia triquetra*	CBS 278.55	MH857481	MH869023	–	–	–	Germany
	JV1410/3	KT281963	KT281975	–	–	–	Czechia
*Onnia tomentosa*	Dai 14806B	KT281965	KT281976	–	–	–	China
	Dai 18900	**OM677241**	**OM677248**	–	**OM937015**	**OM800824**	China
	Dai 22935	**OM677242**	**OM677249**	**ON007278**	**OM937016**	**OM800825**	China
	Dai 23682	OM967277	OM967339	–	–	–	United States
	Dai 23683	**OM677243**	**OM677250**	**OM007279**	**OM937017**	**OM80082**	United States
	Dai 23685	OM967279	OM967341	–	–	–	United States
	Vampola 2010	KT281966	KT281977	–	–	–	Czechia
	FP-100585-5p	KF996516	–	–	–	–	Canada
	OT-Slu	KF996518	–	–	–	–	Sweden
	T. Niemela 9079	MF319075	MF319006	–	–	–	Finland
	SFC20170810-01	MT044403	–	–	–	–	Russia
	Cui 9986	KT281964	–	–	–	–	China
	HHB-18573	KT955001	–	–	–	–	United States
	LOO-13789-Q	KF996517	–	–	–	–	United States
	TW 445		AF311023	–	–	–	Germany
*Phellinopsis conchata*	L7601	KU139188	KU139257	–	–	–	United States
*Phellinopsis andina*	MR 1203	KP347542	KP347528	–	–	–	Argentina
*Phellinus igniarius*	85-917	AY340048	AF311027	–	–	–	Germany
*Porodaedalea chinensis*	Cui 10252	KX673606	MH152358	–	MH101479	MG585301	China
*P*. *pini*	No-6170-T	JX110037	JX110081	–	–	JX109993	United States
	FP102111T	JX110036	JX110080	–	–	–	United States
*P*. *yunnanensis*	Dai 3072	MG585282	MH152380	–	–	MG585292	China
*Ramaria rubella*	AFTOL 724	AY854078	AY645057	–	AY786064	AY883435	United States
*Sanghuangporus sanghuang*	Cui 14419	MF772789	MF772810	MF972246	MF973483	MF977790	China
*Suillus pictus*	AFTOL 717	AY854069	AY684154	AY858965	AY883429	AY883429	–
*Thelephora ganbajun*	ZRL20151295	LT716082	KY418908	KY418987	KY419043	KY419093	China
*Trametes versicolor*	ZRL20151477	LT716079	KY418903	KY418984	KY419041	KY419091	China
*Trechispora alnicola*	AFTOL 665	DQ411529	AY635768	–	–	DQ059052	United States
*Ustilago maydis*	AFTOL 505	AY854090	AF453938	–	AY485636	AY885160	–

*New sequences are in bold; “–” represents missing data.*

Recently, important research advances have been made in the studies of species diversity and divergence times of fungi (He et al., 2019; Varga et al., 2019; Wu et al., 2020; Dai et al., 2021; Wang K. et al., 2021; Zong et al., 2021). At present, more than 140,000 species of fungi were described, accounting for 3.50%–6.04% of an estimate of 2,200,000–3,800,000 (Hawksworth and Lücking, 2017; Wang et al., 2020). Hymenochaetaceae, the core family of wood-inhabiting fungi, recognizes 672 poroid species in the world (Wu et al., 2022). In addition, the determination of the divergence times within Basidiomycota based on fossil evidence has provided a robust set of age estimates for higher taxa (Zhao et al., 2017; He et al., 2019; Wang X. W. et al., 2021), with fossil species such as *Quatsinoporites cranhamii* S.Y. Smith et al. (2004) and Berbee and Taylor (2010) representing a minimum age of 125 Mya for Hymenochaetaceae. Meanwhile, the molecular dating studies of macrofungi widely pay attention to ectomycorrhizal fungi, saprotrophic fungi, and pathogenic fungi (Hibbett and Matheny, 2009; Chen et al., 2015; Song et al., 2016; Truong et al., 2017; Li et al., 2020; Liu et al., 2022; Wang X. W. et al., 2022). A series of studies related to the divergence time of pathogenic fungi, such as *Coniferiporia* L.W. Zhou and Y.C. Dai, *Heterobasidion* Bref., and *Phytophthora ramorum* Werres et al., have been published (Chen et al., 2015; Jung et al., 2021; Wang X. W. et al., 2022). However, divergence times of important coniferous pathogenic fungal *Onnia* have not been well resolved.

In the study of biogeography, the evolution of species is an important issue requiring reconstructing the origin, speciation, and distribution patterns of organisms (Page, 2003; Seehausen et al., 2014). To date, macrofungi, especially wood-inhabiting fungi, being closely interacted with host plants, are an interesting subject in biogeographic research (Chen et al., 2015; Song et al., 2016; Varga et al., 2019; Li et al., 2020; Wang X. W. et al., 2022). For example, the ancestral geographic origin analyses suggested that coniferous pathogenic fungal *Coniferiporia* originated in Asia and then extend to Europe and North America (Wang X. W. et al., 2022). Regrettably, *Onnia*, a crucial member of wood-inhabiting fungi, is very much understudied in this regard.

In this article, a new species from Yunnan Province, China, *Onnia himalayana*, is phylogenetically and morphologically described. Meanwhile, a hypothesis for species diversification and origin of *Onnia* is proposed, namely, species of this genus seem to originate in the coniferous forests of southwest China.

## Materials and Methods

### Sample Collection

Species, voucher specimens, and GenBank accession numbers of *Onnia* used in the present study were obtained from Asia, Europe, and North America. They are listed in Table 1.

### Morphology

The studied *Onnia* specimens are deposited in the herbarium of the Institute of Microbiology, Beijing Forestry University (BJFC). Morphological descriptions are based on field notes and herbarium specimens. Sections were studied at a magnification of up to 1,000 × using a Nikon Eclipse 80i microscope and phase contrast illumination. Microscopic features and measurements were made from slide preparations stained with Cotton Blue and Melzer’s reagent. Basidiospores were measured from sections cut from the tubes. To represent variation in the size of basidiospores, 5% of measurements were excluded from each end of the range and are given in parentheses. In the description: KOH = 5% potassium hydroxide, IKI = Melzer’s reagent, IKI– = neither amyloid nor dextrinoid, CB = Cotton Blue, CB + = cyanophilous in Cotton Blue, CB– = acyanophilous in Cotton Blue, L = arithmetic average of basidiospore length, W = arithmetic average of basidiospore width, *Q* = *L*/*W* ratios, and *n* = number of basidiospores/measured from given number of specimens. Color terms are from Anonymous (1969) and Petersen (1996).

### DNA Extraction, Polymerase Chain Reaction, and Sequencing

Total DNA was extracted from dried specimens with a rapid plant genome extraction kit (Aidlab Biotechnologies Co., Ltd, Beijing, China), modified following Cao et al. (2012) and Zhao and Cui (2013). The internal transcribed spacers (ITS), large subunit of nuclear ribosomal RNA gene (LSU rDNA), partial DNA-directed RNA polymerase II subunit one gene (*rpb1*) and subunit two gene (*rpb2*), and partial translation elongation factor 1-alpha gene (*tef1*α) were amplified with primer pairs ITS 4 (5′-TCC TCC GCT TAT TGATAT GC-3′) and ITS 5 (5′-GGA AGT AAA AGT CGT AAC AAG G-3′; White et al., 1990), LR0R (5′-ACC CGC TGA ACT TAA GC-3′) and LR7 (5′-TAC TAC CAC CAA GAT CT-3′), RPB1-Af (5′-GAR TGY CCD GGD CAY TTY GG-3′) and RPB1-Cf (5′-CCN GCD ATN TCR TTR TCC ATR TA-3′; Matheny et al., 2002), fRPB2-5F (5′-GAY GAY MGW GAT CAY TTY GG-3′) and fRPB2-7cR (5′-CCC ATR GCT TGY TTR CCC AT-3′; Liu et al., 1999; Matheny, 2005), and EF1-1567R (5′-ACH GTR CCR ATA CCA CCS ATC TT-3′) and EF1-983F (5′-GCY CCY GGH CAY CGT CAY TTY AT-3′; Rehner and Buckley, 2005; Matheny et al., 2007), respectively. The polymerase chain reaction (PCR) procedures were as follows: for ITS sequences, an initial denaturation at 95°C for 3 min, followed by 34 cycles at 94°C for 40 s, 54°C for 45 s, 72°C for 1 min, and a final extension of 72°C for 10 min (Zhou et al., 2021a,b); for LSU rDNA region, an initial denaturation at 94°C for 1 min, followed by 34 cycles at 94°C for 30 s, 50°C for 1 min, 72°C for 1.5 min, and a final extension of 72°C for 10 min (Shen et al., 2016); for *rpb1*, *rpb2*, and *tef1*α regions, an initial denaturation at 94°C for 2 min, followed by 10 cycles at 94°C for 40 s, 60°C for 40 s, and 72°C for 2 min, then followed by 37 cycles at 94°C for 45 s, 55°C for 1.5 min, 72°C for 2 min, and a final extension at 72°C for 10 min (Chen et al., 2015; Wang X. W. et al., 2021). Sequencing for PCR products was conducted by BGI Tech Solutions Beijing Liuhe Co., Ltd., Beijing, China. Sequences were assembled and proofread with Geneious (version 9.0.2^1^, accessed 1 May 2021) and then submitted to GenBank under the accession numbers in Table 1.

### Phylogenetic Analyses

All sequences were aligned with AliView (version 3.0; Larsson, 2014) and MAFFT (version 7; Katoh and Standley, 2013), and then manually adjusted. A dataset of 30 specimens composed of ITS + LSU rDNA sequences was subjected to maximum likelihood (ML), maximum parsimony (MP), and Bayesian inference (BI) phylogenetic analyses using RAxML (version 8, Stamatakis, 2014), PAUP (version 4.0b10; Swofford, 2002), and MrBayes (version 3.2.7a; Ronquist et al., 2012), respectively, following Zhao et al. (2021, 2022a,b). The GTRGAMMA model was chosen as the substitution model for ML analysis. Obtained phylograms were viewed with FigTree (version 1.4.4).

### Divergence Time Estimation

In this study, a dataset with 47 specimens (Figure 2) was used to infer the divergences times of species in the genus *Onnia* based on a dataset composed of ITS + LSU rDNA + *rpb1* + *rpb2* + *tef1*α sequences. The divergence times were estimated with BEAST (version 2.6.5; Bouckaert et al., 2014), using two ribosomal RNA genes (ITS and LSU rDNA) and three protein-coding genes (*rpb1*, *rpb2*, and *tef1*α). An XML (Extensible Markup Language) file was generated with BEAUti (version 2). The rates of evolutionary changes at nuclear acids were estimated using ModelTest (version 3.7) with the GTR substitution model (Posada and Crandall, 1998). Divergence time and corresponding CIs were conducted with a log-normal relaxed molecular clock and the Yule speciation prior. Three fossil time points, i.e., *Archaeomarasmius leggettii* Hibbett et al. (1995, 1997), *Quatsinoporites cranhamii* S.Y. Smith et al. (2004) and Berbee and Taylor (2010), and *Paleopyrenomycites devonicus* Taylor et al. (1999, 2005), representing the divergence time at Agaricales, Hymenochaetaceae, and between Ascomycota and Basidiomycota, respectively, were selected for calibration. The offset age with a gamma distributed prior (scale = 20 and shape = 1) was set as 90, 125, and 400 Mya for Agaricales, Hymenochaetaceae, and Basidiomycota, respectively. After 10,000,000 generations, the first 10% were removed as burn-in. The log file was checked for convergence with Tracer (version 1.5^2^). Consequently, a maximum clade credibility (MCC) tree was summarized with TreeAnnotator (version 2.6.5), annotating clades with more than 0.8 posterior probability (PP).

### Inferring Historical Biogeography

Reconstruct Ancestral State in Phylogenies (RASP) (version 4.2) was used to reconstruct historical biogeography for the genus *Onnia* with a dispersal-extinction-cladogenesis (DEC) model (Yu et al., 2015, 2020). For historical biogeographic analyses, the posterior distributions of the dataset (Table 1), including two ribosomal RNA genes (ITS and LSU rDNA) and three protein-coding genes (*rpb1*, *rpb2*, and *tef1*α), were estimated with BEAST. The geographic distributions for *Onnia* were identified in three areas: (A) Asia, (B) Europe, and (C) North America.

## Results

### Phylogeny of *Onnia*

**FIGURE 1:**
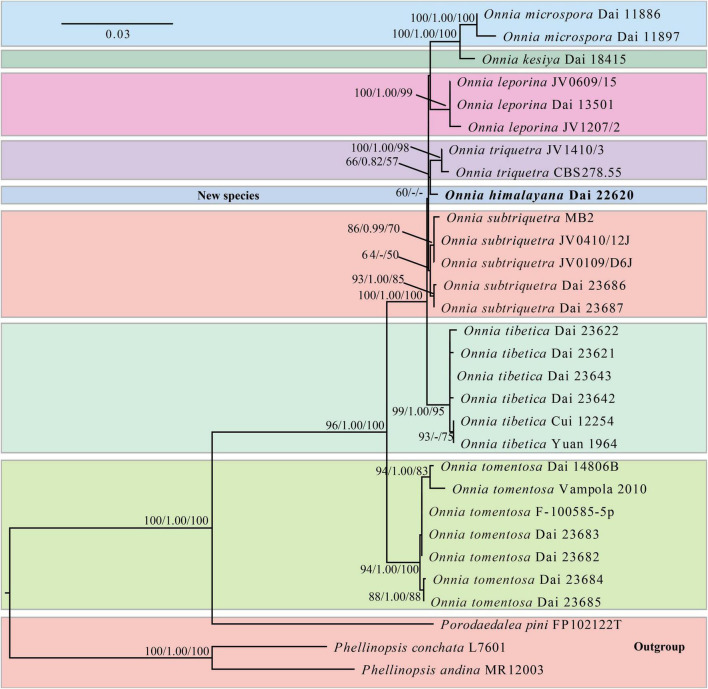
Maximum likelihood (ML) phylogenetic tree of *Onnia* based on ITS and LSU rDNA sequences, with *Porodaedalea pini*, *Phellinopsis conchata*, and *P*. *andina* as outgroups. ML bootstrap values (≥ 50%)/maximum parsimony (MP) bootstrap values (≥ 50%)/Bayesian inference (BI) posterior probabilities (≥ 0.8) of each clade are indicated along branches. A scale bar in the upper left indicates substitutions per site.

The ITS and LSU rDNA sequences are provided in Tables 1, 30 voucher specimens represent eight species of *Onnia*, one species of *Porodaedalea* Murrill, and two species of *Phellinopsis* Y.C. Dai. The dataset had an aligned length of 2,127 characters, including 1,750 constant, 155 parsimony-uninformative, and 222 parsimony-informative characters. MP analysis yielded a tree (tree length = 495, consistency index = 0.8889, homoplasy index = 0.1111, retention index = 0.9073, and rescaled consistency index = 0.8064). The best model of BI for the ITS and LSU rDNA dataset was GTR + I + G, and the average SD of split frequencies was less than 0.01. The topology of the ML tree was chosen to represent the phylogenetic relationship with *Porodaedalea pini* (Brot.) Murrill, *Phellinopsis conchata* (Pers.) Y.C. Dai, and *P*. *andina* (Plank and Ryvarden) Rajchenb. and Pildain as outgroups, since ML, MP, and BI resulted in similar topologies. The result suggests that *O*. *himalayana* is closely related to *O*. *triquetra* (Figure 1).

**FIGURE 2:**
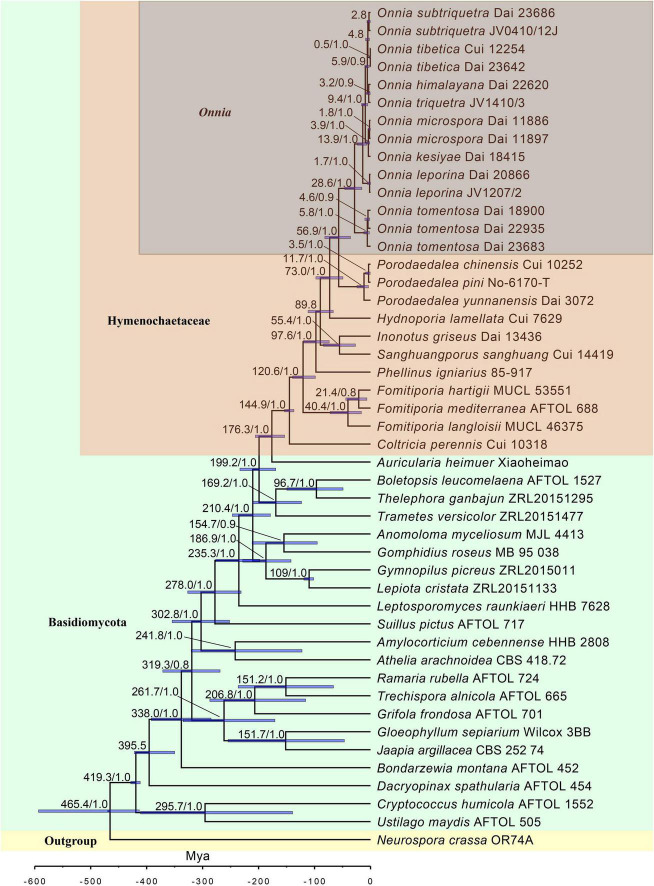
Estimated divergence of *Onnia* generated from molecular clock analyses using a combined dataset of ITS, LSU rDNA, *rpb1*, *rpb2*, and *tef1*α sequences. Estimated mean divergence time (Mya) and posterior probabilities (PP) > 0.8 are annotated at the internodes. The 95% highest posterior density (HPD) interval of divergence time estimates is marked by horizontal blue bars.

### Divergence Time Estimation for *Onnia*

The results of divergence time estimation show (Figure 2) that Hymenochaetaceae emerged earlier with a mean stem age of 176.3 Mya [95% highest posterior density (HPD) of 153.5–205.7 Mya] and a mean crown age of 144.9 Mya (95% HPD of 136.8–153.8 Mya), which is consistent with previous studies (Wang X. W. et al., 2021; Ji et al., 2022). In Hymenochaetaceae, *Onnia* is closely related to the genus *Porodaedalea*, which is most deeply diversified during the Paleogene, with a mean stem age of 56.9 Mya (95% HPD of 35.9–81.6 Mya) and full support (1.0 PP, Figure 2 and Table 2). The majority of species of *Onnia* emerged in the Neogene, especially in the Pliocene. *Onnia tomentosa* is the oldest species with a mean stem age of 28.6 Mya (95% HPD of 15.5–46.2 Mya), while *O*. *triquetra* and *O*. *himalayana* are younger than the other species with a stem age of 3.2 Mya (95% HPD of 0.7–7.1 Mya).

**TABLE 2:** Inferred divergence time of species in the genus *Onnia*.

Genus/Species	Means of stem age (Mya)/95% HPD (Mya)/Posterior probabilities	Means of crown age (Mya)/95% HPD (Mya)/Posterior probabilities
*Onnia*	56.9/35.9–81.6/1.0	28.6/15.5–46.2/1.0
*O*. *tomentosa*	28.6/15.5–46.2/1.0	5.8/2.0–12.0/1.0
*O*. *leporina*	13.9/7.3–23.6/1.0	1.7/0.1–5.6/1.0
*O*. *kesiyae*	3.9/1.4–7.6/1.0	3.9/1.4–7.6/1.0
*O*. *microspora*	3.9/1.4–7.6/1.0	1.8/0.4–4.1/1.0
*O*. *triquetra*	3.2/0.7–7.1/0.9	3.2/0.7–7.1/0.9
*O*. *himalayana*	3.2/0.7–7.1/0.9	3.2/0.7–7.1/0.9
*O*. *tibetica*	4.8/1.9–9.0/–	0.5/0–1.8/1.0
*O*. *subtriquetra*	4.8/1.9–9.0/–	2.8/0.5–6.4/–

*Hyphen “–” represents a posterior probability (PP) < 0.8.*

### The Historical Biogeography of *Onnia*

**FIGURE 3:**
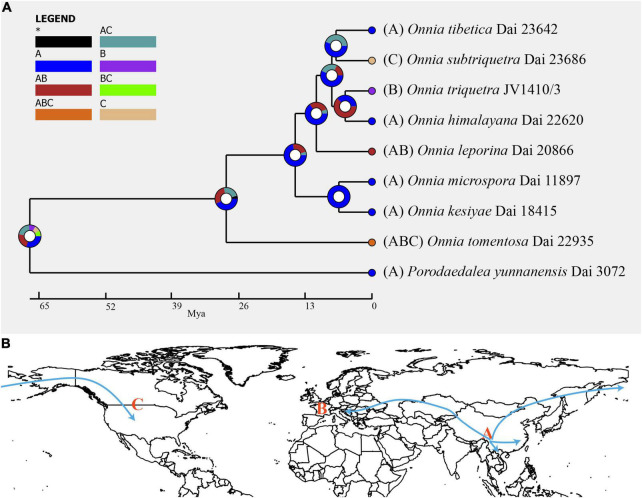
**(A)** Ancestral state reconstruction and divergence time estimation of *Onnia* using a dataset containing ITS, LSU rDNA, *rpb1*, *rpb2*, and *tef1*α sequences. A pie chart at each node indicates the possible ancestral distributions inferred from dispersal-extinction-cladogenesis (DEC) analysis implemented in RASP. A black asterisk represents other ancestral ranges. **(B)** Possible dispersal routes of *Onnia* in the Northern hemisphere. Regions are labeled as follows: (A) Asia, (B) Europe, (C) North America, (AB) Asia and Europe, (AC) Asia and North America, (BC) Europe and North America, and (ABC) Asia, Europe, and North America.

**FIGURE 4:**
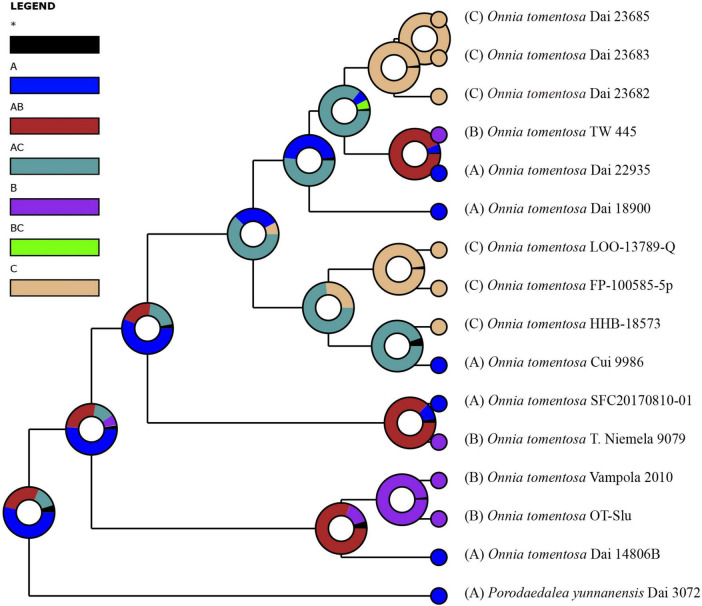
Ancestral state reconstruction and divergence time estimation of *Onnia tomentosa* using a dataset containing ITS and LSU rDNA sequences. A pie chart at each node indicates the possible ancestral distributions inferred from dispersal-extinction-cladogenesis (DEC) analysis implemented in RASP. A black asterisk represents other ancestral ranges. Regions are labeled as follows: (A) Asia, (B) Europe, (C) North America, (AB) Asia and Europe, (AC) Asia and North America, and (BC) Europe and North America.

Inferred historical biogeography scenarios using RASP are shown in Figure 3. The RASP analysis suggests that Asia is the center of origin of the genus *Onnia*, and suggests that five dispersal events (three from Asia to Europe, and two from Asia to North America) and two vicariance (Eurasia and North America) events occurred during the distribution of this genus. Six species are found in Asia, three in Europe, and two in North America, suggesting that Asia is still the center of *Onnia* species. Moreover, there are three species, *O*. *tomentosa*, *O*. *tibetica*, and *O*. *himalayana*, distributed in southwest China, which implies that this region may be a more precise center of origin within Asia. Indeed, a total of 15 specimens of *O*. *tomentosa*, namely, six in North America, five in Asia, and four in Europe, have been collected (Figure 4 and Table 1). The dataset of ancestral state reconstruction suggested that Asia is the ancestral area (Figure 4). Meanwhile, possible concealed dispersal routes were inferred (Figure 3B): (1) Asia to North America and (2) Asia to Europe.

### Taxonomy

**FIGURE 5:**
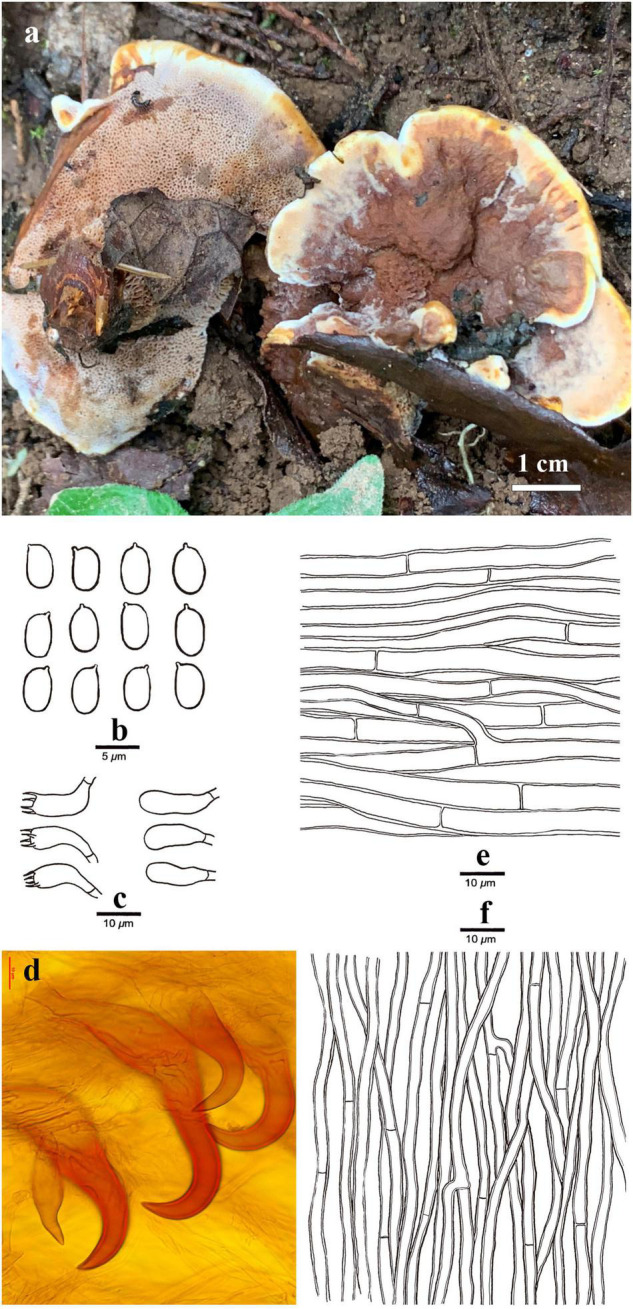
Basidiocarps and microscopic structures of *Onnia himalayana* (Holotype, Dai 22620). **(a)** Basidiocarps of *Onnia himalayana*; **(b)** Basidiospores; **(c)** Basidia and basidioles; **(d)** Hymenial setae; **(e)** Hyphae from upper tomentum; and **(f)** Hyphae from trama.

***Onnia himalayana*** Y.C. Dai, H. Zhao and Meng Zhou, sp. nov. (Figure 5).

*MycoBank*: MB: 844317.

*Type*: CHINA. Yunnan Province, Dali, Cangshan Geopark, on root of *Pinus yunnanensis*, 30 VIII 2021, Dai 22620 (Holotype, BJFC037194).

*Etymology*: *Himalayana* (Lat.), refers to the species being found in the eastern Himalayan area.

Basidiocarps annual, laterally to centrally stipitate, solitary, without odor or taste and corky when fresh, becoming hard corky upon drying. Pilei dimidiate to circular, projecting up to 3 cm, 4 cm wide, and 8 mm thick at the center. Pileal surface clay buff with cream to buff margin, velutinate, and azonate when fresh, becoming cinnamon, homogeneous, distinctly velutinate, and azonate when dry; margin sharp, curving downward when dry. Pore surface clay pink when fresh, becoming fulvous when dry, sterile margin distinct, up to 2 mm wide; pores angular, 3–4 per mm; and dissepiments thin, strongly lacerate to dentate. Context duplex, upper layer fulvous, more or less spongy, up to 4 mm thick, lower layer umber, hard corky, up to 2 mm thick, no demarcation zone between the two layers. Tubes are concolorous with pores, hard corky, and up to 2 mm long. Stipe clay buff, hard corky when dry, velutinate, up to 1 cm long, 8 mm diam; pores decurrent on the stipe.

Hyphal system monomitic, generative hyphae simple septate, IKI–, CB–; tissues darkening but otherwise unchanged in KOH. Context: hyphae in the upper layer are pale yellowish to golden yellow, slightly thick-walled, occasionally branched, frequently simple septate, straight, regularly arranged, and 5–7 μm diam; hyphae in the lower layer are yellowish to golden brown, slightly thick- to thick-walled, occasionally branched, with frequent simple septa, straight, regularly arranged, not agglutinated, and 4–5.5 μm diam; hyphae in stipe similar to those in context. Tubes: Tramal hyphae hyaline to yellowish, thin- to slightly thick-walled, rarely branched, frequently septate, more or less flexuous, subparallel along the tubes, not agglutinated, and 2.5–4.5 μm diam.

Hymenium: Setae hooked, sharply pointed at apex, dark brown, thick-walled, deep-rooting, embedded in trama and projecting from hymenium, and 40–78 × 14–20 μm; cystidia and cystidioles absent; basidia clavate, with four sterigmata and a simple septum at the base, 12–15 × 5–6 μm; and basidioles dominant, in shape similar to basidia, but slightly smaller. Basidiospores ellipsoid to oblong-ellipsoid, hyaline, thin-walled, smooth, IKI–, CB–, 5-6 × 3.2-4 (-4.1) μm, *L* = 5.62 μm, *W* = 3.63 μm, and *Q* = 1.55 (*n* = 30/1).

## Discussion

The discovery of new fungal species has rapidly increased with the development of molecular techniques, drawing attention to the huge fungal diversity that exists on earth (Cui et al., 2019; He et al., 2019; Wu et al., 2020, 2022; Dai et al., 2021; Wang K. et al., 2021; Zhang and Dai, 2021; Ji et al., 2022). Hymenochaetaceae is a core family of macrofungi that consists of approximately 670 poroid species (Wu et al., 2022) and is an interesting subject for species diversity studies (Dai, 2010; Wu et al., 2020, 2022; Dai et al., 2021; Wang X. W. et al., 2022). Although *Onnia* is a small genus in this family, some species of *Onnia* are important pathogenic fungi that cause Tomentosus Root Rot on trees of *Picea* and *Pinus* (Hunt and White, 1998; Germain et al., 2009; Dai, 2010; Ji et al., 2017). As species distribution of *Onnia* is usually closely related to host trees (Ji et al., 2017; Wu et al., 2022), the genus is ideal for studying species diversity, divergence times, and biogeography.

Currently, dating analyses have provided a deep insight into the evolution of macrofungi using multigene analyses (Zhao et al., 2017; He et al., 2019; Varga et al., 2019). Our analysis of divergence times using a dataset of two ribosomal RNA genes (ITS and LSU rDNA) and three protein-coding genes (*rpb1*, *rpb2*, and *tef1*α) suggests that *Onnia* and *Porodaedalea* possibly emerged in the Paleogene with a mean stem age of 56.9 Mya (95% HPD of 35.9–81.6 Mya) and full support (1.0 PP; Figure 2 and Table 2). Considering the divergence estimation of Pinaceae (206 Mya) and the fossil record of Hymenochaetaceae (125 Mya), this estimation of *Onnia* and *Porodaedalea* seems reasonable (Smith et al., 2004; Berbee and Taylor, 2010; Magallón et al., 2015; Ran et al., 2018). Moreover, the basal modern species, *O*. *tomentosa*, occurred in 28.6 Mya, which is consistent with the timing of the second pulse of rapid uplift of the Qinghai-Tibet Plateau (between 20 and 30 Mya; Wang et al., 2012, 2018). Most species of *Onnia* emerged about 5 Mya (Figure 2 and Table 2), i.e., late Miocene to Pliocene, and adapted to a low temperature, facilitating survival in the Quaternary Ice Age.

Biogeographic studies of macrofungi have been very successful for ectomycorrhizal fungi, such as *Amanita* (see Sánchez-Ramírez et al., 2015; Truong et al., 2017), saprotrophic *Lentinula* (see Hibbett et al., 1998), and pathogenic fungi, e.g., *Heterobasidion* (Chen et al., 2015) based on molecular analyses. Our results suggest that the species distribution of *Onnia* has a distinct biogeographical pattern, similar to other wood-decaying fungi (Sato et al., 2017; Han et al., 2018; Li et al., 2020). Species of *Onnia* appear to have originated in Asia, especially in the Hengduan-Himalayan region which is a global biodiversity hotspot, and this conclusion supports previous studies on the origination of wood-decaying fungi (Song et al., 2016; Li et al., 2020; Wang X. W. et al., 2022). Three species, *O*. *himalayana*, *O*. *tibetica*, and *O*. *tomentosa*, occur in the Hengduan-Himalayan region. The basal species, *O*. *tomentosa*, emerged at 28.6 Mya (Figure 2 and Table 2), and maybe dispersal occurred between East Asia and North America *via* the Beringia (Bering Land Bridge). However, a vicariance event, such as the opening of the Bering Strait, could limit gene flow and species dispersal in the Old World and the New World (Hibbett, 2001; Cai et al., 2014; Li et al., 2020).

## Conclusion

In this study, our dataset of divergence times suggests that *Onnia* and *Porodaedalea* possibly emerged in the Paleogene. Most species of *Onnia* emerged in the late Miocene to Pliocene and adapted to a low temperature, and therefore survived in the Quaternary Ice Age. Species appear to have originated in the coniferous forests of southwest China, then spread across the Northern Hemisphere with host plants. Geographic separation led to a diversification of new species in the Old World and New World. A total of nine species are recognized, namely, eight species that grow on gymnosperms and one species that grows on angiosperms. Furthermore, a new species, *Onnia himalayana*, is proposed and illustrated based on phylogenetic and morphological evidence.

## Data Availability Statement

All the sequences have been deposited in GenBank; the accession numbers are listed in Table 1.

## Author Contributions

HZ: data analyses, formal analyses, conceived the ideas, and original draft and review. MZ: data curation and the draft of new species. X-YL: review and editing. FW: project administration and review and editing. Y-CD: funding acquisition, investigation, description of new species, and review and editing. All authors have read the manuscript.

## Conflict of Interest

The authors declare that the research was conducted in the absence of any commercial or financial relationships that could be construed as a potential conflict of interest.

## Publisher’s Note

All claims expressed in this article are solely those of the authors and do not necessarily represent those of their affiliated organizations, or those of the publisher, the editors and the reviewers. Any product that may be evaluated in this article, or claim that may be made by its manufacturer, is not guaranteed or endorsed by the publisher.

## Footnotes

1
http://www.geneious.com

2
http://beast.bio.ed.ac.uk/Tracer
